# Safety and Immunogenicity of a Tetravalent Dengue DNA Vaccine Administered with a Cationic Lipid-Based Adjuvant in a Phase 1 Clinical Trial

**DOI:** 10.4269/ajtmh.17-0416

**Published:** 2018-01-22

**Authors:** Janine R. Danko, Tadeusz Kochel, Nimfa Teneza-Mora, Thomas C. Luke, Kanakatte Raviprakash, Peifang Sun, Monika Simmons, James E. Moon, Rafael De La Barrera, Luis Javier Martinez, Stephen J. Thomas, Richard T. Kenney, Larry Smith, Kevin R. Porter

**Affiliations:** 1 Infectious Diseases Directorate, Naval Medical Research Center, Silver Spring, Maryland;; 2 Walter Reed Army Institute of Research, Silver Spring, Maryland;; 3 ClinReg Biologics, LLC, Brisbane, California;; 4 Vical Incorporated, San Diego, California

## Abstract

We conducted an open label, dose escalation Phase 1 clinical trial of a tetravalent dengue DNA vaccine (TVDV) formulated in Vaxfectin^®^ to assess safety and immunogenicity. A total of 40 dengue- and flavivirus-naive volunteers received either low-dose (1 mg) TVDV alone (*N* = 10, group 1), low-dose TVDV (1 mg) formulated in Vaxfectin (*N* = 10, group 2), or high-dose TVDV (2 mg, group 3) formulated in Vaxfectin^®^ (*N* = 20). Subjects were immunized intramuscularly with three doses on a 0-, 30-, 90-day schedule and monitored. Blood samples were obtained after each immunization and various time points thereafter to assess anti-dengue antibody and interferon gamma (IFNγ) T-cell immune responses. The most common adverse events (AEs) across all groups included mild to moderate pain and tenderness at the injection site, which typically resolved within 7 days. Common solicited signs and symptoms included fatigue (42.5%), headache (45%), and myalgias (47.5%). There were no serious AEs related to the vaccine or study procedures. No anti-dengue antibody responses were detected in group 1 subjects who received all three immunizations. There were minimal enzyme-linked immunosorbent assay and neutralizing antibody responses among groups 2 and 3 subjects who completed the immunization schedule. By contrast, IFNγ T-cell responses, regardless of serotype specificity, occurred in 70%, 50%, and 79% of subjects in groups 1, 2, and 3, respectively. The largest IFNγ T-cell responses were among group 3 subjects. We conclude that TVDV was safe and well-tolerated and elicited predominately anti-dengue T-cell IFNγ responses in a dose-related fashion.

## INTRODUCTION

Recent publications suggest that the global impact of dengue infections is greater than that previously published by the World Health Organization. An estimated 96 million apparent infections and an additional 294 million inapparent infections occur worldwide annually.^1^ There are four serologically distinct dengue RNA viruses designated DENV-1, DENV-2, DENV-3, and DENV-4. Complications from acute infection can lead to hospitalization, debilitation, and death. An effective dengue vaccine is a high priority for countries where the disease is endemic, and for travelers and military populations that frequently travel to endemic regions. We have pursued the nucleic acid immunization approach to develop a candidate tetravalent dengue vaccine. Toward this goal, a prototype monovalent dengue-1 DNA vaccine construct (D1ME) containing the premembrane (prM) and envelope (E) genes of dengue-1 WestPac was evaluated in a Phase 1 clinical trial and determined to be safe but poorly immunogenic and did not produce a robust neutralizing antibody response.^2^ T-cell interferon gamma (IFNγ) responses, however, were much more pronounced.

Vaxfectin^®^ adjuvanted plasmids have been used to enhance the humoral responses of other DNA vaccines.^3–5^ Vaxfectin is a cationic lipid:neutral lipid combination adjuvant compound.^6^ A nonhuman primate (NHP) vaccine study was conducted using this adjuvant formulated with our tetravalent dengue DNA vaccine (TVDV). Rhesus monkeys were given three intramuscular (IM) doses on days 0, 28, and 84 and subsequently challenged with live dengue virus 6 months after the initial dose. The use of Vaxfectin significantly increased the anti-dengue neutralizing antibody responses and provided significant protection against a dengue-2 virus challenge.^7^ Based on these results, a tetravalent DNA vaccine with and without Vaxfectin was studied in a Phase 1 clinical trial in dengue-seronegative healthy volunteers. The results of this clinical trial are described in this article.

## MATERIALS AND METHODS

### TVDV and the Vaxfectin-formulated vaccine.

The TVDV is a mixture of equal amounts of four monovalent double-stranded plasmid DNA vaccines produced under current Good Manufacturing Practices conditions in the United States. Each monovalent plasmid contains the prM and E genes of dengue 1, 2, 3, or 4 viruses cloned into the backbone plasmid VR1012 (Vical Incorporated, San Diego, CA). The derivative virus strains and further information about these monovalent vaccines were previously described.^7^ Because the antecedent NHP study with TVDV used IM injections, this route of administration was the chosen method of vaccine delivery for the human trial. Additional support for this method of delivery was derived from a Vaxfectin-adjuvanted plasmid DNA influenza vaccine candidate tested in human trials, which was also delivered as an IM injection and induced favorable humoral responses.^8^ At the time of this study, the maximum amount of DNA that could be formulated in a 1 mL volume of Vaxfectin was 1 mg (Lot #0690043 TVDV was used). All components of the vaccine were frozen until the day of dosing and were completely thawed at room temperature for a minimum of 2 hours (not to exceed 4 hours) before formulation as per manufacturer’s specifications. A 1 mL Vaxfectin lipid suspension (Lot # 0909855; Vical Incorporated) was prepared using dried lipid vials and 0.9% sterile sodium chloride (Lot #01090014; Vical Incorporated). The formulated vaccine was administered within 8 hours of formulation. All vaccine injections were 1 mL in final volume. As described in the following section, some subjects received only the TVDV. For those receiving TVDV and Vaxfectin, 0.7 mL of the suspended adjuvant was added to the TVDV per study-specific procedures, and 1 mL of the formulated vaccine was administered to each subject in one of the deltoid muscles. Given that the maximum amount of DNA that could be formulated in a 1 mL volume of Vaxfectin was 1 mg, group 3 subjects received two injections for each dose (one injection in each deltoid).

### Clinical trial study design and safety endpoints.

The study objectives were to evaluate the safety, tolerability, and immunogenicity of TVDV with and without Vaxfectin in healthy adult subjects. This was a dose escalating, open-label study. Healthy civilian and active duty military volunteers (age 18–50 years inclusive) were recruited through the Walter Reed Army Institute of Research Clinical Trials Center, which is co-located with the Naval Medical Research Center (NMRC) and where all study procedures were performed. Recruitment was conducted by non-coercive means and according to current Good Clinical Practice guidelines. The NMRC Institutional Review Board, along with the U.S. Army Human Subjects Research Review Board, reviewed and approved the study protocol (NMRC.2011.0012) in compliance with all applicable federal regulations governing the protection of human subjects. Before conducting any study procedure, an informed consent document was signed by each subject.

Flavivirus serologies were performed to assess for preexisting antibody to DENV1–4, Japanese encephalitis virus (JEV), West Nile virus, and yellow fever (YF) virus by enzyme-linked immunosorbent assay (ELISA) initially, and followed by DENV1–4 and JEV plaque reduction neutralization tests (PRNT) of those negative by ELISA. Subjects who had detectable anti-dengue and other flavivirus antibodies at screening were excluded to avoid interference with interpretation of dengue serological responses. Individuals with planned travel to dengue endemic areas during the study and those with known autoimmune conditions or anti-nuclear antibody titers > 1:80 at screening were also excluded, as well as women who were breastfeeding or pregnant. Once determined to be eligible, subjects were enrolled sequentially into one of three groups: Group 1 subjects (*N* = 10) received 1 mg of TVDV at each dosing; group 2 subjects (*N* = 10) received 1 mg of TVDV formulated with Vaxfectin; and group 3 subjects (*N* = 20) received 2 mg total of TVDV formulated with Vaxfectin (1 mg administered in each upper extremity). After the initial vaccination, each subject received a second and third dose on study days 30 and 90, respectively. To evaluate anti-dengue antibody and cellular immune responses, blood samples were obtained before each vaccination and monthly thereafter, up until day 270.

The final clinical visit was at study day 270. Safety was monitored by medical history, physical examination, review of laboratory results, adverse events (AEs), and memory aid information. The safety and tolerability measures used in this study were the occurrence of local and systemic AEs, serious AEs (SAEs), and changes in clinical laboratory tests or vital signs. Monitoring for safety following each vaccination included assessing each subject for local and systemic reactions, reviewing each subject’s symptom memory aid (diary), and performing targeted physical examinations. Solicited AEs were obtained for 7 days following each vaccination and overall AEs were captured through day 180. A telephone follow-up was conducted at study day 360 to assess for safety.

### Measurement of anti-dengue antibody responses.

Indirect ELISA tests were performed in duplicate with a positive control in every assay and conducted as previously described.^9,10^ Briefly, microtiter plates were coated with purified dengue virus antigen and incubated overnight at 4°C and then blocked with 5% nonfat dry milk in phosphate buffered saline/Tween 20 at 37°C for 1 hour. Serum samples, diluted in blocking buffer, were added to the plates, incubated for 1 hour at 37°C, washed, and the plates reacted with peroxidase-conjugated goat anti-human immunoglobulin G (IgG). Following another wash, the plates were reacted with 2,2’-Azino-bis(3-ethylbenzothiazoline-6-sulfonic acid) substrate to detect bound antibody.

The ELISA assay was used as a screening tool to monitor study subjects for anti-dengue humoral immune responses. Therefore, serum samples were tested only at a screening dilution of 1:100. The net optical density (OD) values measured at 405 nm were determined by subtracting the absorbance of the test serum with negative control antigen from the absorbance of test serum with the DENV antigen. The cutoff value for seropositivity was set at an OD of ≥ 0.10 because the mean OD value, plus 2 standard deviations (SD) for negative control sera, was consistently below this value.

Anti-dengue neutralizing antibody in serum was assayed using a high throughput dengue ELISA microneutralization (MN) test as previously described.^10^ The results are expressed as reciprocal MN50 titers, which represent the reciprocal serum dilution giving a 50% reduction in absorbance readout when compared with a virus dose control lacking serum. MN50 titers < 10 are considered negative and titers ≥ 10 as positive. For the results to be comparable with the results from the previous clinical trial of the monovalent dengue 1 DNA vaccine, MN50 was used to determine neutralization capacity.

### IFNγ enzyme-linked immunospot (ELISPOT) assays for measurement of cell-mediated immunogenicity (CMI).

T-cell IFNγ responses were determined using an ELISPOT assay as previously described with the following modifications.^2^ IFNγ responses were quantitatively measured at pre-vaccination (day 0) and at 30 days after the first, second, and third dose of TVDV (day 30, day 60, and day 120). Each sample was tested on four peptide (15–20 mers, overlapping by 10–11 aa) pools of serotype-specific E proteins representing each of the four dengue serotypes and two peptide pools for serotypes 1 and 2 prM proteins. Because the peptides were prepared in dimethyl sulfoxide (DMSO), a DMSO control was used as the negative control for determination of baseline response values. The mitogen phytohemagglutinin (PHA) was used as the positive control. DMSO control wells and antigen wells were plated with 2 × 10^5^ peripheral blood mononuclear cells (PBMCs) per well. For the PHA control, PBMCs were plated at 6 × 10^4^ cells/well.

Most samples were run in triplicates, but 13% were run in duplicates. Only the positive control wells were run in single wells. The average of the duplicates or triplicates was used. The approach taken to remove outliers was to determine the mean ±2 SD for each triplicate measurement. If one value was outside of this range, it was removed from the analysis. There were 38 outliers among the total 5,771 data entries; therefore, the rate for outliers was 0.66%. The spot counts were expressed as spot forming units (SFUs) per 10^6^ PBMCs for the summary statistics. A peptide-specific response was defined as the mean value of the peptide wells minus the mean value of the negative control wells. A sample was considered to have a positive dengue antigen–specific response if the mean response value in peptide wells was at least 2-fold higher than that of the control wells and had at least 50 SFU/10^6^ cells. If no control value was available, no result was calculated. If the control value was zero, it was set to “1” to perform the fold-increase calculation. If a positive response was seen to one or more peptide pools at day 0, the subject was considered to have preexisting CMI, and hence was excluded from further data analysis.

### Data analysis.

The primary study endpoints were safety and tolerability as measured by the rates for AEs. The rates for AEs were compared by time point and group. The grading scale for local and systemic reactogenicity was as follows: mild interference with daily activities (grade 1), moderate or some interference with daily activities (grade 2), significant or severe interference preventing daily activities (grade 3), and potentially life threatening or resulting in an emergency room visit or hospitalization (grade 4).

Vaccine-related events (probably or possibly-related) were tabulated by study group. For categorical variables, the number and percentage of patients in each category are summarized. Continuous variables are summarized with the number of observations (*n*), mean, SD, median, minimum, and maximum values. As this was a pilot study, no statistical comparisons between groups were planned because of the small sample size and anticipated high variability between subjects. Missing data are reflected by varying sample sizes across visits. The primary endpoints of safety and secondary endpoints of immunogenicity were analyzed for all subjects who received at least one injection of vaccine.

## RESULTS

### Study participants and demographics.

**Figure 1.**
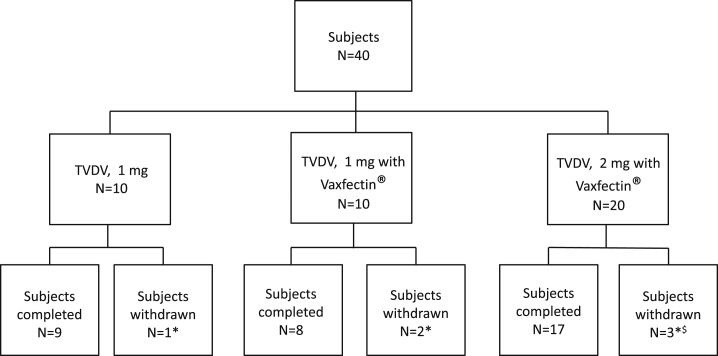
Study subject disposition. Blocks indicate the total number of subjects in each group and the disposition of such subjects during the study. * Lost to follow-up because of relocation, $ Consent withdrawn (*N* = 1) after first dose but safety data available.

A total of 40 healthy flavivirus-naive adult subjects (age 18–50 years inclusive) were enrolled in the study and received at least one study vaccination. Ten subjects in group 1 each received low dose TVDV (1 mg) without Vaxfectin and 10 subjects in group 2 each received low dose TVDV (1 mg) formulated in Vaxfectin. Group 3 consisted of 20 subjects who received high dose TVDV (2 mg) formulated in Vaxfectin (Figure 1). Table 1 summarizes the demographics of the study subjects. The overall mean age was 34.3 years (range 20.7–50.8) and the largest percentage of subjects (45%) was between 20 and 29 years old. The mean ages for subjects in groups 1, 2, and 3 were 33.4, 38.4, and 32.6, respectively. Most of the participants were Caucasian (42.5%) or African-American (47.5%), and 22 of the subjects were male (55%).

**Table 1:** Demographic characteristics at baseline

	TVDV* (1 mg)	TVDV* (1 mg) with Vaxfectin	TVDV* (2 mg) with Vaxfectin	Total
	(*N* = 10)	(*N* = 10)	(*N* = 20)	(*N* = 40)
Gender (%)				
Male	5 (50)	8 (80)	9 (45)	22 (55)
Age at screening (years) mean (±SD)†	33.4 (9.3)	38.4 (9.7)	32.6 (10.6)	34.3 (10.1)
Race (%)				
Caucasian	5 (50)	5 (50)	7 (35)	17 (42.5)
African-American	3 (30)	4 (40)	12 (60)	19 (47.5)
Asian	1 (10)	1 (10)	0 (0)	2 (5)
Other	1 (10)	0 (0)	1 (5)	2 (5)
Weight (lbs) mean (±SD)†	196.0 (45.5)	191.2 (26.2)	180.0 (32.5)	186.8 (34.7)

* TVDV = tetravalent dengue DNA vaccine.

† SD = standard deviation.

### Safety and reactogenicity endpoint analysis.

All subjects received at least one dose of vaccine. Nine subjects in group 1, eight subjects in group 2, and 17 subjects in group 3 received all three doses. Across all three groups, three subjects were lost to follow-up, two relocated, and one withdrew consent after the first dose because of lack of sustained interest to participate. No subject discontinued study participation because of AEs related to vaccination.

Table 2 lists the definitions of solicited local and systemic reactions. There were no SAEs related to vaccination or study procedures. The most common AEs across each group included pain (31/40, 77%) and tenderness (26/40, 65%) at the injection site (Table 3); these were all graded as mild or moderate and these local symptoms resolved within 7 days for most of the subjects. There were no severe local or systemic reactions observed. Rash was not observed. Of the solicited signs and symptoms, the most commonly experienced were fatigue (17/40, 42.5%), headache (18/40, 45%), and myalgias (19/40, 47.5%). Grade 1 increases in the serum creatinine possibly related to vaccination were observed in one subject in group 2 and three subjects in Group 3. This was observed within the first 2 days following vaccination and lasted for a range of 4 to 91 days (or median of 17.5); and resolved by day 180. In group 3, grade 1 decreases in the serum hemoglobin (*N* = 6), neutrophil count (*N* = 5), and white blood cell count (*N* = 7) judged to be possibly related to vaccination were observed.

**Table 2:** Definitions of solicited local and systemic reactions

Adverse reactions	Definitions
Solicited local site reactions	
Pain	Mild: Does not interfere with activity
Tenderness	Moderate: Repeated use of non-narcotic pain reliever for > 24 hours or interferes with activity
	Severe: Any use of narcotic pain reliever or prevents daily activity
Erythema	Mild: 2.5–5 cm and does not interfere with activity
Induration	Moderate: 5.1–10 cm or interferes with activity
Development of a nodule	Severe: > 10 cm or prevents daily activity
Solicited systemic reactions fever	Mild: 38.0–38.4°C
	Moderate: 38.5–38.9°C
	Severe: 39.0–40.0°C
Headache	Mild: Does not interfere with activity
Myalgias	Moderate: Some interference with activity
Fatigue	Severe: Significant; prevents daily activity
Abdominal pain	
Arthralgias	
Anorexia	
Photophobia	
Eye pain	
Rash	
Nausea	Mild: Does not interfere with activity or ≤ 2 episodes/24 hours
Vomiting	Moderate: Some interference with activity or > 2 episodes/24 hours.
	Severe: Significant; prevents daily activity

**Table 3:** Solicited local and systemic adverse events considered to be at least possibly related to receipt of the vaccination by group, dose, and severity

Reactogenicity	TVDV* (1 mg)				TVDV* (1 mg) with Vaxfectin				TVDV* (2 mg) with Vaxfectin			
	Dose 1 *N* = 10 *n* (%)	Dose 2 *N* = 10 *n* (%)	Dose 3 *N* = 10 *n* (%)	Total *N* = 10 *n* (%)	Dose 1 *N* = 10 *n* (%)	Dose 2 *N* = 10 *n* (%)	Dose 3 *N* = 9 *n* (%)	Total *N* = 10 *n* (%)	Dose 1 *N* = 20 *n* (%)	Dose 2 *N* = 19 *n* (%)	Dose 3 *N* = 17 *n* (%)	Total *N* = 20 *n* (%)
Local reactions												
Pain												
None	5 (50)	4 (40)	10 (100)	3 (30)	2 (20)	1 (10)	0 (0)	0 (0)	4 (20)	3 (16)	1 (6)	1 (5)
Mild	5 (50)	5 (50)	0 (0)	6 (60)	3 (30)	6 (60)	7 (78)	4 (40)	7 (35)	7 (37)	1 (6)	2 (10)
Moderate	0 (0)	1 (10)	0 (0)	1 (10)	5 (50)	3 (30)	2 (22)	6 (60)	9 (45)	9 (47)	15 (88)	17 (85)
Severe	0 (0)	0 (0)	0 (0)	0 (0)	0 (0)	0 (0)	0 (0)	0 (0)	0 (0)	0 (0)	0 (0)	0 (0)
Erythema												
None	10 (100)	10 (100)	10 (100)	10 (100)	10 (100)	9 (90)	7 (78)	7 (70)	20 (100)	19 (100)	16 (94)	19 (95)
Mild	0 (0)	0 (0)	0 (0)	0 (0)	0 (0)	0 (0)	2 (22)	2 (20)	0 (0)	0 (0)	1 (6)	1 (5)
Moderate	0 (0)	0 (0)	0 (0)	0 (0)	0 (0)	1 (10)	0 (0)	1 (10)	0 (0)	0 (0)	0 (0)	0 (0)
Severe	0 (0)	0 (0)	0 (0)	0 (0)	0 (0)	0 (0)	0 (0)	0 (0)	0 (0)	0 (0)	0 (0)	0 (0)
Induration												
None	10 (100)	10 (100)	10 (100)	10 (100)	5 (50)	8 (80)	5 (56)	5 (50)	20 (100)	19 (100)	15 (88)	18 (90)
Mild	0 (0)	0 (0)	0 (0)	0 (0)	4 (40)	2 (20)	4 (44)	4 (40)	0 (0)	0 (0)	2 (12)	2 (10)
Moderate	0 (0)	0 (0)	0 (0)	0 (0)	1 (10)	0 (0)	0 (0)	1 (10)	0 (0)	0 (0)	0 (0)	0 (0)
Severe	0 (0)	0 (0)	0 (0)	0 (0)	0 (0)	0 (0)	0 (0)	0 (0)	0 (0)	0 (0)	0 (0)	0 (0)
Tenderness												
None	7 (70)	7 (70)	10 (100)	5 (50)	4 (40)	2 (20)	1 (11)	1 (10)	4 (20)	3 (16)	0 (0)	1 (5)
Mild	3 (30)	3 (30)	0 (0)	5 (50)	5 (50)	4 (40)	7 (78)	4 (40)	9 (45)	9 (47)	5 (29)	6 (30)
Moderate	0 (0)	0 (0)	0 (0)	0 (0)	1 (10)	4 (40)	1 (11)	5 (50)	7 (35)	7 (37)	12 (71)	13 (65)
Severe	0 (0)	0 (0)	0 (0)	0 (0)	0 (0)	0 (0)	0 (0)	0 (0)	0 (0)	0 (0)	0 (0)	0 (0)
Systemic Reactions												
Fever												
None	10 (100)	9 (90)	10 (100)	8 (80)	10 (100)	10 (100)	9 (100)	10 (100)	18 (90)	17 (89)	17 (100)	17 (85)
Mild	0 (0)	0 (0)	0 (0)	1 (10)	0 (0)	0 (0)	0 (0)	0 (0)	2 (10)	2 (11)	0 (0)	3 (15)
Moderate	0 (0)	1 (10)	0 (0)	1 (10)	0 (0)	0 (0)	0 (0)	0 (0)	0 (0)	0 (0)	0 (0)	0 (0)
Severe	0 (0)	0 (0)	0 (0)	0 (0)	0 (0)	0 (0)	0 (0)	0 (0)	0 (0)	0 (0)	0 (0)	0 (0)
Headache												
None	9 (90)	8 (80)	9 (90)	6 (60)	7 (70)	8 (80)	8 (89)	6 (60)	14 (70)	13 (68)	11 (65)	9 (45)
Mild	1 (10)	1 (10)	0 (0)	2 (20)	3 (30)	1 (10)	1 (11)	3 (30)	4 (20)	4 (21)	2 (12)	5 (25)
Moderate	0 (0)	1 (10)	1 (10)	2 (20)	0 (0)	1 (10)	0 (0)	1 (10)	2 (10)	2 (11)	4 (23)	6 (30)
Severe	0 (0)	0 (0)	0 (0)	0 (0)	0 (0)	0 (0)	0 (0)	0 (0)	0 (0)	0 (0)	0 (0)	0 (0)
Arthralgias												
None	10 (100)	9 (90)	10 (100)	9 (90)	9 (90)	9 (90)	8 (89)	9 (90)	17 (85)	17 (90)	16 (94)	15 (75)
Mild	0 (0)	0 (0)	0 (0)	0 (0)	1 (10)	1 (10)	1 (11)	1 (10)	3 (15)	1 (5)	0 (0)	3 (15)
Moderate	0 (0)	1 (10)	0 (0)	1 (10)	0 (0)	0 (0)	0 (0)	0 (0)	0 (0)	1 (5)	1 (6)	2 (10)
Severe	0 (0)	0 (0)	0 (0)	0 (0)	0 (0)	0 (0)	0 (0)	0 (0)	0 (0)	0 (0)	0 (0)	0 (0)
Myalgias												
None	8 (80)	6 (60)	10 (100)	6 (60)	5 (50)	10 (100)	6 (67)	5 (50)	13 (65)	13 (68)	11 (65)	10 (50)
Mild	1 (10)	2 (20)	0 (0)	1 (10)	3 (30)	0 (0)	3 (33)	3 (30)	1 (5)	4 (21)	1 (6)	2 (10)
Moderate	1 (10)	2 (20)	0 (0)	3 (30)	2 (20)	0 (0)	0 (0)	2 (20)	6 (30)	2 (11)	5 (29)	8 (40)
Severe	0 (0)	0 (0)	0 (0)	0 (0)	0 (0)	0 (0)	0 (0)	0 (0)	0 (0)	0 (0)	0 (0)	0 (0)
Fatigue												
None	9 (90)	7 (70)	10 (100)	6 (60)	7 (80)	8 (80)	8 (89)	6 (60)	13 (65)	13 (68)	11 (65)	11 (55)
Mild	1 (10)	2 (20)	0 (0)	3 (30)	3 (30)	2 (20)	0 (0)	3 (30)	5 (25)	4 (21)	3 (18)	4 (20)
Moderate	0 (0)	1 (10)	0 (0)	1 (10)	0 (0)	0 (0)	1 (11)	1 (10)	2 (10)	2 (11)	3 (18)	5 (25)
Severe	0 (0)	0 (0)	0 (0)	0 (0)	0 (0)	0 (0)	0 (0)	0 (0)	0 (0)	0 (0)	0 (0)	0 (0)
Nausea												
None	10 (100)	10 (100)	10 (100)	10 (100)	9 (90)	10 (100)	8 (89)	9 (90)	17 (85)	18 (95)	14 (82)	15 (75)
Mild	0 (0)	0 (0)	0 (0)	0 (0)	1 (10)	0 (0)	1 (11)	1 (10)	3 (15)	0 (0)	3 (18)	4 (20)
Moderate	0 (0)	0 (0)	0 (0)	0 (0)	0 (0)	0 (0)	0 (0)	0 (0)	0 (0)	1 (5)	0 (0)	1 (5)
Severe	0 (0)	0 (0)	0 (0)	0 (0)	0 (0)	0 (0)	0 (0)	0 (0)	0 (0)	0 (0)	0 (0)	0 (0)

* TVDV = tetravalent dengue DNA vaccine.

### Vaccine-induced antibody responses (ELISA and MN).

No anti-DENV IgG antibody responses were detectable by ELISA in group 1 subjects as determined by screening ELISA. Three of the 10 subjects in group 2 had measurable ELISA antibody responses; however, only one showed tetravalent responses (D1–4). These ELISA antibody responses were present at day 180 and persisted to study day 270. In group 3, five subjects had ELISA antibody detected against one, three, or all four dengue virus serotypes between days 120 and 270. Anti-dengue ELISA antibodies against all four serotypes were seen in two group 3 volunteers starting on day 90 and persisting through day 270.

Low-level neutralizing antibodies to DENV-1 were seen in one volunteer (group 3) on day 120 and a tetravalent response in another volunteer on day 180. None of the other subjects had measurable anti-dengue neutralizing antibodies across all time points as measured by the MN test.

### Vaccine-induced T-cell responses.

CMI against dengue E and prM protein peptide pools was measured using the IFNγ ELISPOT assay and the data are summarized in Table 3. Overall, four subjects from group 2 and five subjects from group 3 were excluded because of pre-existing CMI. After exclusion of these subjects, CMI response rates were calculated as the total number of positive responders divided by the total number of subjects included for the analysis. The total response rates regardless of serotype-specificity were 70%, 50%, and 79% for groups 1, 2, and 3, respectively (Table 4).

**Table 4:** Interferon-gamma results

	Number of subjects tested and included	Number of subjects tested but excluded*	Total positive responders† number (%)	Response to numbers of serotypes number (%)												Response range‡ (SFU/10^6^ PBMCs)
				After first dose				After second dose				After third dose				
				1 ST§	2 ST	3 ST	4 ST	1 ST	2 ST	3 ST	4 ST	1 ST	2 ST	3 ST	4 ST	
Group 1	10	0	7 (70)	2 (20)	3 (30)	3 (30)	3 (30)	2 (20)	2 (20)	2 (20)	4 (40)	2 (20)	4 (40)	0 (0)	4 (40)	50–223
Group 2	6	4	3 (50)	1 (17)	0 (0)	0 (0)	0 (0)	2 (33)	0 (0)	0 (0)	0 (0)	2 (33)	1 (17)	0 (0)	0 (0)	56–98
Group 3	14	5	11 (79)	2 (14)	3 (21)	4 (28)	2 (14)	1 (7)	2 (14)	2 (14)	3 (21)	2 (14)	4 (28)	0	4 (28)	50–531

PBMC = peripheral blood mononuclear cell; SFU = spot forming unit.

* Subjects who had positive response(s) ≥ 1 peptide pools at Day 0 were excluded in the summary.

† A positive responder was counted if response to ≥ 1 peptide pool was seen during the period of day 30 to day 120.

‡ The range of SFU within the group was counted as the range between the lowest and the highest response at any time to any antigen.

§ Serotype.

**Figure 2.**
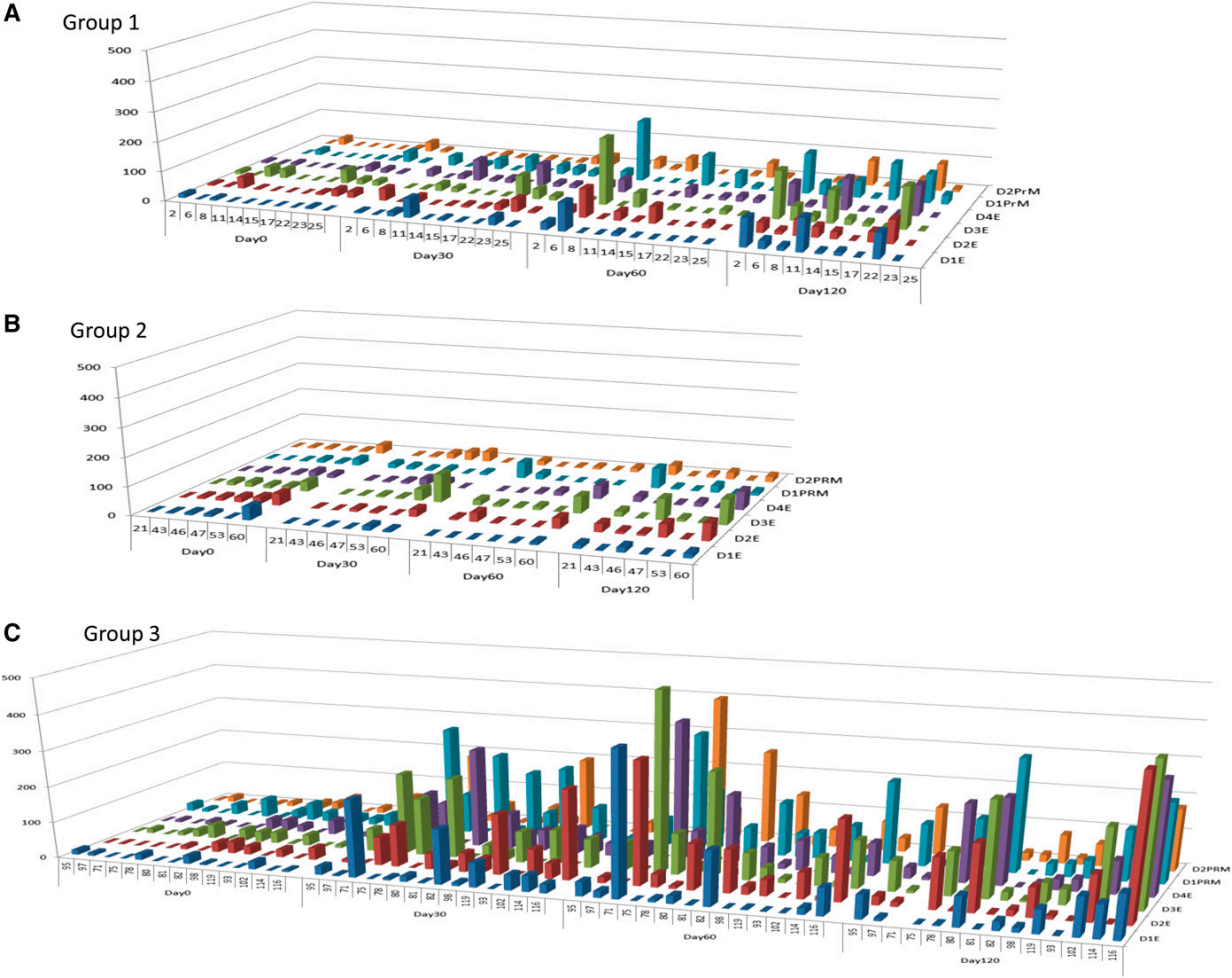
Interferon gamma T-cell enzyme-linked immunospot (ELISPOT) results. Each graph represents ELISPOT results for subjects who received either 1 mg total of TVDV alone (**A**, group 1), 1 mg TVDV with Vaxfectin (**B**, group 2) or 2 mg with Vaxfectin (**C**, group 3). The individual volunteer numbers are listed on the *x* axis, group by day. The *y* axis shows ELISPOT result in number of spots/10^5^ total cells. The *z* axis shows the serotype-specific peptide pool used to stimulate the cells. Each bar represents the average ELISPOT result for the indicated volunteer for the indicated peptide pool.

CMI response rates were also compared based on the dosage group and serotype-specific peptide pool reactivity (Figure 2). The magnitude of IFNγ CMI responses are also shown, with the results expressed as SFU per 10^5^ total cells. Group 3 had the highest IFNγ CMI responses compared with the other groups.

## DISCUSSION

The aim of dengue vaccine development programs is to produce a candidate vaccine that elicits solid long-lasting protective immune responses against all four dengue serotypes to reduce the incidence of symptomatic infection. Although anti-dengue neutralizing antibodies are capable of protecting against dengue infection, there is growing consensus that the optimal tetravalent dengue vaccine should generate long-lasting neutralizing antibodies as well as T-cell responses against all four dengue serotypes. Because dengue live–attenuated vaccines induce these types of immune responses, theoretically they should provide the best protection. However, immune interference resulting from the tetravalent components giving rise to an imbalanced immune response remains a concern. Several dengue vaccine approaches have been tested in human clinical trials and there are numerous candidates in preclinical development.^11^

The relative ease of manufacturing, unique stability, and non-replicating properties make plasmid DNA immunization an attractive platform for developing a tetravalent dengue vaccine. An earlier Phase 1 clinical trial of a prototype monovalent dengue-1 DNA vaccine showed the vaccine to be well tolerated and capable of generating good anti-dengue IFNγ T-cell responses, but poor anti-dengue neutralizing antibody responses. To enhance the humoral response to dengue, we explored using the DNA vaccine formulated in the proprietary adjuvant, Vaxfectin. NHP testing of TVDV-Vaxfectin demonstrated immunity consisting of increased anti-dengue neutralizing antibody responses compared with TVDV alone. As expected, T-cell responses were also generated, but the use of Vaxfectin resulted in no significant improvement of the T-cell responses compared with TVDV without Vaxfectin.

The study reported here describes the evaluation of the TVDV-Vaxfectin combination in a Phase 1 human clinical trial. The results demonstrate the safety and tolerability of the TVDV-Vaxfectin formulation, with minimal side effects. Anti-dengue IFNγ T-cell responses to the vaccine were generated in nearly 80% of subjects receiving the highest dose. These response rates were comparable with those seen in the high-dose group of the monovalent dengue-1 DNA vaccine clinical trial. The addition of Vaxfectin to TVDV (at the 1 mg dose) did not significantly improve the T-cell response rates. When comparing the IFNγ T-cell responses of all three groups, there was no significant difference in the rate of response, but there appeared to be a dose-dependent trend toward increased magnitude of response as evidenced by the higher IFNγ ELISPOT values in the 2 mg TVDV-Vaxfectin dose group.

Historically, criteria for immunological success in dengue vaccine clinical trials have been based on neutralizing antibody responses. However, Sanofi’s clinical end-point efficacy trial of their chimeric YF-dengue virus (CYD) vaccine sparked controversy (concerns) because despite high anti-dengue neutralizing antibody GMTs for the four DENV serotypes after vaccination, the clinical efficacy was 30.2% (95% confidence interval = 13.4–56.6) and varied by serotype.^12^ The in vitro neutralizing antibodies measured in a vaccine subgroup did not predict the overall in vivo clinical experience following infection. Subsequent clinical trials of the CYD vaccine candidate, including two Phase 3 placebo-controlled trials in pediatric populations in Asia and Latin America have demonstrated higher levels of vaccine efficacy against symptomatic, virologically confirmed dengue (between 56.5% and 60.8%), but there are still questions about what types of protective immunity responses the vaccine are providing and the best test to measure levels of protective antibodies.^13,14^ In a study of 48 individuals with serologically confirmed symptomatic dengue by Sirivichayakul et al.,^15^ nine with high preexisting PRNT_50_ titers to subsequent infecting dengue serotypes still developed symptomatic infections. Continued exploration and characterization of the cellular immune responses following natural dengue virus infections (primary and beyond) with comparison to the same responses following vaccination are needed.^16–21^ Some authors have shown that the addition of plasmid DNA as a prime or boost to other vaccine constructs results in robust polyfunctional T-cells and higher antigen-specific central memory CD8 + T-cells suggesting long-term memory responses.^21^ Whether anti-dengue T-cell immunity alone is sufficient to provide protection against dengue remains to be determined. Despite animal data showing that passive transfer of anti-dengue T-cells can protect against dengue virus challenge, there are no definitive human studies that conclusively show anti-dengue cellular immunity alone provides significant protection against dengue infection. Accordingly, although the TVDV generates high CMI responses, improvement in anti-dengue neutralizing antibody responses is highly desired for optimal protection.

There is evidence, however, that a T-cell-based viral vaccine is capable of providing some level of protection against infection. Lillie et al.^22^ published a phase 2a human clinical trial where adult volunteers were immunized with a single injection of a modified vaccinia virus Ankara-vectored vaccine that expressed conserved influenza nucleoprotein and matrix proteins. All subjects before challenge had HI titers of ≤ 1:10 to the challenge virus. The vaccine provided significant protection against laboratory-confirmed influenza following intranasal challenge with live virus as reflected by significantly reduced symptoms and viral shedding. Whether these results translate to dengue or other flaviviruses remains to be determined, but the data do show that T-cell-based vaccines can play a role in altering influenza disease manifestations.

Anti-dengue neutralizing antibody responses were lacking in most of the subjects. In light of the TVDV NHP test results, this was unexpected and suggests that the adjuvant Vaxfectin offered little benefit to TVDV when administered to humans by the IM needle route at the doses used in the Phase 1 clinical trial. These suboptimal neutralizing antibody responses may be attributable either to the adjuvant’s lack of efficacy in humans, a suboptimal dose, or to a suboptimal delivery method.

The previous clinical trial evaluating the monovalent dengue DNA vaccine showed that a 1 mg dose delivered by Biojector showed no measurable antibody levels. The higher dose of D1ME DNA vaccine (5 mg) induced detectable neutralizing antibodies in 41% of subjects at 2 months after the third vaccine dose (day 252). The highest DNA vaccine dose administered during the TVDV study was 2 mg total, 0.5 mg per serotype construct, which was lower than that which elicited a modest immune response in the D1ME phase 1 clinical trial. Ideally, we wanted to use a total DNA vaccine dose of 5 mg, but the maximum dose of TVDV was limited by the highest concentration and dose that could be formulated with Vaxfectin. At the time of this clinical trial, formulation of Vaxfectin^®^ with higher concentrations had not been completed. Now that higher doses can be formulated (4 mg DNA/1 mg Vaxfectin per injection), it is feasible to test higher vaccine doses for improved immune responses. The D1ME and TVDV clinical trial outcomes suggested a dose response relationship between DNA vaccine and humoral immune responses. From this, we postulate that a higher dose of TVDV-Vaxfectin might stimulate greater levels of neutralizing antibodies.

A suboptimal vaccine delivery method may have also contributed to the poor neutralizing antibody responses. A recently published article highlights how the site and delivery method of DNA vaccine influence the induced immune response.^23^ The NHP study demonstrated excellent humoral responses from TVDV-Vaxfectin when administered by the IM route using Biojector 2000, a needle-free injection device actuated by CO_2_. However, this route of administration was not selected for the TVDV Phase 1 clinical trial based on data from a clinical study of the H5N1 influenza vaccine adjuvanted with Vaxfectin.^8^ This vaccine trial showed no significant differences between humoral immune responses among subjects who received H5N1 influenza vaccine with Vaxfectin IM by needle injection and those who received the same vaccine via IM Biojector. The difference in vaccine delivery methods may have contributed to the discordance between the outcomes of the TVDV-Vaxfectin NHP study and Phase 1 human clinical trial. Because the NHP study did not include a study arm involving needle IM injection, we cannot conclude with certainty that differences in administration device played a role in suboptimal neutralizing antibody responses in humans. Preclinical studies are underway to explore alternative DNA vaccine delivery methods and include needle-free jet injection and electroporation.

After reviewing the available literature on DNA vaccine administration, delivering the TVDV by needle injection was deemed a logical approach to evaluate the primary end point of safety for the Vaxfectin-formulated TVDV vaccine. Another driving factor was that delivering the vaccine by needle instead of Biojector, which at the time was a relatively new technology, would be more conducive to mass vaccination campaigns in developing countries. Given the results of the current trial and with advances in jet injection devices, a follow-on study administering the vaccine by jet injection should be considered.

In conclusion, TVDV-Vaxfectin was safe and well tolerated in this early Phase 1 human clinical trial. Whereas anti-dengue IFNγ T-cell responses occurred in most of the study subjects, anti-dengue neutralizing antibody responses were poor. Utilization of alternative delivery methods as well as examining prime-boost approaches may result in a more robust and long-lasting humoral immune response.
